# Preparation and Evaluation of Collagen-Based Patches as Curcumin Carriers

**DOI:** 10.3390/polym12102393

**Published:** 2020-10-17

**Authors:** Zoi Terzopoulou, Anna Michopoulou, Artemis Palamidi, Elena Koliakou, Dimitrios Bikiaris

**Affiliations:** 1 Laboratory of Chemistry and Technology of Polymers and Dyes, Department of Chemistry, Aristotle University of Thessaloniki, GR 541 24 Thessaloniki, Greece; artemispalamidi@gmail.com (A.P.); dbic@chem.auth.gr (D.B.); 2 Biohellenika Biotechnology Company, Leoforos Georgikis Scholis 65, GR 555 35 Thessaloniki, Greece; annamichop@yahoo.gr; 3 Laboratory of Histology and Embryology, Medical School, Aristotle University of Thessaloniki, GR 541 24 Thessaloniki, Greece; elenakoliakou@gmail.com

**Keywords:** collagen, psoriasis, curcumin, skin patches, chitosan nanoparticles

## Abstract

Patients with psoriasis are dissatisfied with the standard pharmacological treatments, whether systemic or topical, with many of them showing interest in complementary and alternative medicine. Curcumin (Cur), a natural polyphenol derived from turmeric, has recently gained attention for skin-related diseases because of its proven anti-inflammatory action. However, topical treatment with Cur would be inadequate because of its hydrophobicity, instability, and low bioavailability. In addition, hyperkeratosis and lack of moisture in psoriatic skin result in low penetration that would prevent actives from permeating the stratum corneum. In this work, a polymer-based formulation of Cur for the topical treatment of psoriasis is reported. To improve the physicochemical stability of Cur, it was first encapsulated in chitosan nanoparticles. The Cur-loaded nanoparticles were incorporated in a hydrophilic, biocompatible collagen-based patch. The nanoparticle-containing porous collagen patches were then chemically cross-linked. Morphology, chemical interactions, swelling ratio, enzymatic hydrolysis, and Cur release from the patches were evaluated. All patches showed excellent swelling ratio, up to ~1500%, and after cross-linking, the pore size decreased, and their hydrolysis rates decelerated. The in vitro release of Cur was sustained with an initial burst release, reaching 55% after 24 h. Cur within the scaffolds imparted a proliferation inhibitory effect on psoriatic human keratinocytes in vitro.

## 1. Introduction

Topical and transdermal drug delivery systems are gaining growing attention in the field of pharmaceutical technology. Avoiding the oral route and delivering active compounds through the skin enhances their therapeutic effect and pharmacological properties [1]. The materials used to prepare drug delivery patches are predominately polymers, either natural or synthetic, and are considered to be the backbone of topical delivery systems [2]. Natural polymers and their hydrogels are used to fabricate patches because of their desirable characteristics, like hydrophilicity, biocompatibility, swelling ability, and controlled degradation rate that in turn controls the drug release rate. Their hydrophilicity also helps with permeation by hydrating the skin [3].

To enhance the penetration of drugs through the skin but to also allow the loading of lipophilic active agents, nanosized carriers are being used [4]. Chitosan nanoparticles (CS-NPs) stand out in transdermal formulations [5,6,7,8,9]. CS is a naturally derived polymer, well established as a drug carrier owning biocompatibility, low immunogenicity, biodegradability, mucoadhesivity, in situ gelation, permeation enhancement, and inherent antimicrobial activity [10,11]. Recently, various polymeric nano-/microparticle hydrogel composites were recognized as superior local drug delivery vehicles because they can yield composite and nanocomposite hydrogels with tailored properties [12,13]. In these hybrid systems, where the hydrogel is formed in a nanoparticle suspension, the nanoparticles contain the active compound to sustain its release and improve its skin penetration, and the hydrogels stabilize the nanoparticles and provide the necessary hydrophilicity [12].

Curcumin (Cur) is a polyphenol of natural origin isolated from turmeric that is characterized as “GRAS” (generally recognized as safe) by the U.S. Food and Drug Administration [6]. Cur shows anti-inflammatory, antioxidant, anticancer, and wound healing properties [14]. Numerous formulations for the topical delivery of Cur have been reported [15,16,17,18,19], focusing on its wound healing properties. Cur is a complementary medicine proven to be efficient against various skin diseases, including psoriasis, with robust evidence from randomized clinical trials [20]. Because of its the hydrophobicity, low bioavailability, and chemical instability, a variety of advanced formulations to improve its therapeutic efficacy have been reported [21,22,23,24,25] and recently reviewed [26,27,28]. The most recent ones include nanoemulsions [21,29], nanohydrogels [22], nanoparticles [25,30] with a focus on CS [5] and nanoparticles in hydrogels [31,32], nanostructured lipid carriers [33], lipid-based self-nanoemulsifying systems [34], and ethosomes [24]. Cur-loaded CS-NPs have superior permeation through Strat-M membranes and mouse skin [6] and significantly accelerate wound healing rates [5,8]. Recently, to prolong the effectiveness of Cur for transdermal delivery applications, it was loaded in ethosomes that had improved inflammatory effect in comparison with pure Cur [18], in composite membranes of cellulose with piperine to enhance its skin permeation [35], and in patches of hydroxymethyl propyl cellulose with acceptable sustained release rates [36]. Composite films of carrageenan/locust bean gum/montmorillonite with dispersed Cur had slow release rates and accelerated wound healing [37]. A nanofiber patch prepared by electrospinning with polycaprolactone and polyethyleneglycol proved promising as Cur carriers in a preliminary in vitro evaluation, as they showed good entrapment and release values [38].

About 80% of the patients with psoriasis use some form of conventional topical treatment, which usually suffers from low drug penetration that is further restricted because of the hyperkeratosis and lack of moisture of the psoriatic skin [39]. Since the inhibitory activity of Cur on potassium channel subtype Kv1.3 was reported [40], more and more studies that examined the potential use of Cur with successful action against psoriasis have emerged. To achieve adequate penetration of the stratum corneum of psoriatic skin to deliver the drugs to the basal layer of the epidermis, nanocarriers are being increasingly explored [28]. Among the studied formulations, a nanogel loaded with Cur and caffeine [41], a nanohydrogel loaded with Cur [22], and a nanoemulsion with imiquimod and Cur [42] showed antipsoriatic efficiency in mouse psoriatic models. NPs of Cur with CS and alginate showed an inhibitory effect on TNF-α-induced psoriatic proliferation of keratinocytes [25].

Because the drugs need to remain at the psoriatic site for a long time, nanoparticle/hydrogel composites that will keep the NPs on the affected area during the release of the active ingredient are the newest approach for the topical treatment of psoriasis. A few of these nanohybrid patches that are made of a hydrogel containing Cur-NPs were reported previously, mainly for wound healing applications, which offered controlled Cur release, decrease in inflammation and acceleration of wound healing, enhanced re-epithelialization, and antimicrobial activity [43,44,45,46,47]. Cur-loaded micelles were added in supramolecular hydrogels that were effective against dermatitis [48]. Cryostructurates of collagen containing Cur lipid NPs were examined as wound healing agents with large porosity, swelling ratio, and slow Cur release over 25 days [47]. Cur–gelatin microspheres in collagen/cellulose nanocrystal composite scaffolds accelerated wound healing and released Cur slowly in a sustained manner [49]. In another approach, Cur that was formulated in a scaffold composed of graphene oxide functionalized with collagen exhibited faster wound healing than collagen itself [50]. A transdermal composite hydrogel based on Carbomer 934P and propylene glycol with Cur–cyclodextrin nanosponges significantly increased the photostability and skin permeation of Cur [51]. When tested specifically against psoriasis, PLGA Cur-NPs incorporated in Carbopol hydrogel [31] and polylysine Cur-NPs in silk fibroin hydrogel [32] had promising results as potential treatment formulations, as they improved the bioactivity of Cur and released it in a controlled manner, enhanced its penetration through the skin, and increased the contact time of the drug with the skin as well as its hydration.

In this study, Cur-loaded CS-NPs were prepared and incorporated in collagen-based patches, and examined for the first time as a potential complementary topical formulation for use on psoriatic skin. Among the hydrogels used as wound dressings and topical drug carriers, collagen proteins are the most common as they not only are a biocompatible and biodegradable component of the human extracellular matrix (ECM) but also actively participate in the wound healing process [52,53]. Their excellent hydrophilicity makes them a suitable candidate for patches with applications in psoriasis, as an adequate amount of water is essential for the successful penetration of drugs during topical delivery [54]. Their biological instability makes their modification, usually by cross-linking, a necessity [55]. Chemical cross-linking with N-(3-dimethylaminopropyl)-N’-ethylcarbodiimide (EDC) and N-hydroxysuccinimide (NHS) gives nontoxic and biocompatible collagen, while addition of the glycosaminoglycan heparin in the cross-linking reaction results in improved in vivo blood biocompatibility [56]. Therefore, the prepared hydrogel composite patches were chemically cross-linked with EDC/NHS and EDC/NHS/heparin to improve their stability and biocompatibility. The physicochemical properties of the CS-NPs and the final CS-NP-containing composite patches were evaluated, including their chemical structure, morphology, swelling ratio, enzymatic hydrolysis rate, and Cur release rates. Finally, their inhibitory effect on the in vitro growth of psoriatic human keratinocytes was evaluated.

## 2. Materials and Methods 

### 2.1. Materials

Chitosan (CS) with high molecular weight (MW: 350,000 g/mol, deacetylation degree > 75%, and viscosity of 1 wt% solution in 1% acetic acid 800–2000 cps at 25 °C), triphenyl phosphate (TPP) used as ionic cross-linking agent, poly(vinyl pyrrolidone) (PVP), and collagen type I from bovine Achilles tendon were supplied by Sigma-Aldrich, St. Louis, MO, USA. Curcumin (Cur) powder was purchased from Fluorochem, UK. Collagenase type I from *Clostridium histolyticum* was purchased from EMD Millipore, MA, USA. All other chemicals were purchased from Sigma-Aldrich and were of analytical grade. 

### 2.2. Synthesis of Curcumin-Loaded Chitosan Nanoparticles

Chitosan nanoparticles (CS-NPs) were prepared with the ionic gelation method, as described by Karri et al. [46]. The mass ratio of CS/TPP was 3/1. Cur was dissolved in ethanol at a concentration of 800 μg/mL. A 0.5 wt% CS solution in 2% *v*/*v* aqueous acetic acid was prepared, and its pH was adjusted to 5 with 2M NaOH. Afterwards, an aqueous solution of 1% *w*/*v* PVP was added in the CS solution to a final PVP concentration of 0.001%. Then, 10 mL of the Cur solution was added in the mixture until a final Cur concentration of 0.1%, followed by the dropwise addition of aqueous TPP solution 0.125% *w*/*v* under constant magnetic stirring (1000 rpm) as the nanoparticles formed spontaneously. Non-entrapped drug was removed by ultracentrifugation at 13,000 rpm for 15 min and resuspension of the nanoparticles, first, with ethanol and, finally, with water. The purified nanoparticles were dispersed with a probe sonicator, frozen, and lyophilized to obtain the Cur-loaded CS-NPs (CS-Cur-NPs). The same procedure but without Cur was followed to prepare the NPs without Cur. 

### 2.3. Preparation of Collagen Patches Loaded with Chitosan Nanoparticles

A total of three different types of collagen patches were prepared: neat collagen patch (Coll), collagen patch cross-linked with EDC/NHS (Coll-Cross), and collagen patch cross-linked with EDC/NHS/heparin (Coll-Cross-Hep). For the preparation of Coll patches, collagen I was dissolved in 0.2 M acetic acid at a concentration of 1 wt% in an ice bath to avoid denaturation. The heterogeneous solution was subjected to mechanical stirring with simultaneous short-term sonication using a probe sonicator to break down the aggregates. Afterwards, the viscous solution was homogenized with an Ultra-Turrax homogenizer for 5 min. Finally, centrifugation at 3500 rpm for 5 min took place to remove air bubbles and insoluble aggregates. The resulting solution was placed in glass beakers, refrigerated, and finally lyophilized with a ScanVac CoolSafe freeze dryer (LaboGene, Allerød, Denmark) at −60 °C and a pressure of 0.25 hPa to yield the collagen I patches, denoted as Coll. 

The lyophilized Coll I patches were cross-linked with EDC/NHS. Briefly, cross-linking took place in a 4-morpholineethanesulfonic acid (MES) 0.05 M buffer solution with pH = 5.4 to minimize EDC hydrolysis. The Coll I patches were initially washed with MES buffer and then immersed in an NHS/EDC solution in MES with NHS/EDC molar ratio = 0.4. The reaction was left to proceed for 2.5 h and was stopped with the addition of a 0.1 M Na_2_HPO_4_ solution. The patches were washed with deionized water, frozen, and lyophilized as described above to yield the patches denoted as Coll-Cross.

Immobilization of heparin (Hep) was performed on the Coll-Cross patches. For this, Coll-Cross patches were immersed in 0.05 M MES buffer for 30 min, followed by the addition of Hep at a final concentration of 1% *w*/*v*. To activate, the carboxyl groups of NHS/EDC at a molar ratio of 0.6 to Hep were added. The reaction took place for 2 h, followed by termination with 0.1 M Na_2_HPO_4_ solution and washing with 4 M NaCl and deionized water. The resulting patches were lyophilized and are denoted as Coll-Cross-Hep.

For the preparation of collagen patches loaded with CS-NPs, the NPs were dispersed in a small amount of water (5 mg/mL) and mixed with the homogeneous solution of soluble collagen (prepared as described above) at a final NP concentration of 10% *w*/*w* in relation to the initial mass of collagen used. The dispersion was sonicated and stirred gently for 45 min to form a hazy yellowish mixture without agglomerates. The patch was obtained with lyophilization as described above. Hybrid Coll-CS-NP patches were then cross-linked with NHS/EDC and NHS/EDC/Hep, respectively, according to the protocols described above. Finally, three CS-NP-loaded patches were prepared: (i) Coll-Cross-CS Cur, (ii) Coll-Cross-Hep, and (iii) Coll-Cross-Hep-CS Cur.

### 2.4. Isolation and Expansion of Human Keratinocytes and Fibroblasts from Skin Biopsies of Patients with Psoriasis

For the isolation of psoriatic human keratinocytes and fibroblasts from the skin of Caucasian patient donors aged 35–53 years, punch biopsies of 5 mm diameter were performed after their informed consent, in accordance with the Declaration of Helsinki. After examination of the protocol and the informed consent documents, the procedure was approved by the ethical standards of the Committee of Bioethics and Ethics (9/12.7.2019) of the Aristotle University of Thessaloniki. The activity of psoriasis in full thickness samples was monitored by histological analysis after staining with hematoxylin and eosin. Fibroblasts and keratinocytes were isolated using the explant method as optimal for the isolation of adequate numbers of cells from small biopsies. The epidermis was separated from the dermis by dissection after treatment with 0.25% trypsin (BIOSERA, # LM-11720/100) for 2 h at 37 °C. For the isolation of the fibroblasts, the pieces of the dermis were placed in culture in DMEM with L-glutamine (BIOWEST, #L0104-500) supplemented with 10% fetal bovine serum (FBS) and penicillin–streptomycin (BIOWEST, # L0018-100) and maintained at 37 °C in a humified atmosphere containing 5% CO_2_. After migration of the fibroblasts from the dermal explants, these cells were amplified in culture and stored in liquid nitrogen as frozen aliquots until use. The epidermis was placed with the basal membrane towards the bottom of a new culture plate. It was cut into very small pieces and maintained in DermaLife K basal medium (CELLSYSTEMS, # LL-004) supplemented with DermaLife K LifeFactors kit containing L-glutamine, apotransferrin, rh TGF-α, rh insulin, hydrocortisone, epinephrine, pituitary extract, and gentamicin/amphotericin included in the kit (CELLSYSTEMS, # LS-1030) at 37 °C in a humified atmosphere containing 5% CO_2_ to let the keratinocytes migrate from the explants. The keratinocytes were then frozen in liquid nitrogen until use for the present cell study experiments.

### 2.5. Characterization 

#### 2.5.1. Characterization of Curcumin-Loaded Chitosan Nanoparticles

Nanoparticle yield, loading capacity (LC), and drug encapsulation efficiency (EE) were calculated with the following equations:

NP yield (%) = (weight of NP) × 100/(initial weight of Cur)(1)

LC (%) = (weight of Cur in NP) × 100/(weight of NP)(2)

EE (%) = (weight of Cur in NP) × 100/(initial weight of Cur)(3)

The weight of curcumin in the NPs was calculated by measuring the amount of the non-entrapped curcumin in the supernatants after the centrifugation step with a Shimadzu HPLC–UV system. The column used was a reverse-phase C-18 (CNW Technologies Athena C18, 120 A, 5 μm, 250 × 4.6 mm), and the mobile phase consisted of 60/40 (*v*/*v*) acetonitrile with 0.1% *v*/*v* phosphoric acid and ultrapure water. Cur was detected at 430 nm. 

Particle size distribution of the prepared nanoparticles was determined by dynamic light scattering (DLS) using a Zetasizer Nano instrument (ZEN 3600; Malvern Instruments, Malvern, Worcestershire, UK) operating with a 532 nm laser. A suitable amount of the NPs was dispersed in distilled water to a final concentration of 1% and was ultrasonicated before the measurement. For each sample, three measurements were conducted.

Fourier transform infrared spectroscopy (FTIR) spectra were obtained using a model Spectrum One FTIR spectrometer (PerkinElmer, Waltham, MA, USA).

X-ray diffraction (XRD) measurements of the samples were performed using a MiniFlex II XRD system from Rigaku Co. (Tokyo, Japan), with CuKα radiation (λ = 0.154 nm) in the angle (2*θ*) range from 5 to 65 degrees.

Differential scanning calorimetry (DSC) measurements were performed with a Pyris 6 instrument (PerkinElmer, Waltham, MA, USA) calibrated with indium and zinc standards, under N_2_ flow. For each measurement, 5–10 mg of each sample was placed in a sealed aluminum pan and heated from ambient temperature to 100 °C with a heating rate of 10 °C/min.

For the in vitro release studies, a dissolution apparatus, DISTEK Dissolution Apparatus Evolution 4300, equipped with an autosampler was used with the USP Apparatus 1 (basket) method. The dissolution medium was acetate buffer CH_3_COOH/CH_3_COONa with pH = 5.5 at a temperature of 32 °C to reflect the normal skin surface temperature and pH [57]. Cur was quantified with a Shimadzu HPLC–UV system. The column used was a reverse-phase C-18 (CNW Technologies Athena C18, 120 A, 5 μm, 250 × 4.6 mm), and the mobile phase consisted of 60/40 (*v*/*v*) acetonitrile with 0.1% *v*/*v* phosphoric acid and ultrapure water. Cur was detected at 430 nm.

#### 2.5.2. Characterization of Collagen Patches

SEM, FTIR, DSC, and XRD measurements were performed on the patches as described in Section 2.5.1.

The swelling ratio was measured using simulated body fluid (SBF) (pH = 7.4). SBF solution was prepared according to Kokubo and Takadama [58]. Each patch was carefully weighed (W_1_) and immersed in SBF at 37 °C. The remnants of materials were wiped off excess surface water using filter paper and weighed (W_2_) at different time intervals (20, 40, 60, 80, 110, and 140 min) until constant weight. The swelling ratio was calculated at different time intervals using the following equation:

Swelling ratio % = (W_2_ − W_1_)/W_1_ × 100 (4)

The enzymatic hydrolysis rate of the patches was evaluated by measuring their mass loss before and after soaking in SBF that contained 4000 ng/mL collagenase type I at pH = 7.4 and a temperature of 37 °C. After predetermined time intervals, each patch was removed from the medium, washed with deionized water, and lyophilized. The mass loss % was calculated with the equation:

Mass loss % = (W_final_ − W_initial_)/W_initial_ × 100 (5)

Cur release from the patches was performed as described for the CS-NPs in Section 2.5.1, but using paddles instead of baskets, according to the UPS29 method with Apparatus 2 (paddle over disk) [59,60]. This method is recommended for simulating transdermal release in vitro. A stainless steel disk assembly was used to hold the patches at the bottom of the vessels, with 25 ± 2 mm distance between the paddle and the disk. The release surface was parallel with the bottom of the paddle blade. Cur was quantified with a Shimadzu HPLC–UV system. The column used was a reverse-phase C-18 (CNW Technologies Athena C18, 120 A, 5 μm, 250 × 4.6 mm), and the mobile phase consisted of 60/40 (*v*/*v*) acetonitrile with 0.1% *v*/*v* phosphoric acid and ultrapure water. Cur was detected at 430 nm.

The fibroblasts and keratinocytes used for cell studies were isolated from the skin biopsies of donors with psoriasis vulgaris after their informed consent according to the protocol described above. Then, they were expanded, and only low passage cells (<2 passages) were used for the present experiments. For the cytocompatibility assays, each scaffold was first sterilized by contact with 70% ethanol overnight, rinsed in a sterile PBS solution, and equilibrated for another 24 h in DMEM or DermaLife K culture medium depending on the subsequent seeding with fibroblasts or keratinocytes, respectively. The fibroblasts were seeded at 5000 on top of each type of collagen scaffold without curcumin placed within 96-well plates and were subjected to the MTT3-(4,5-Dimethyl-2-thiazolyl)-2,5-diphenyl-2H-tetrazolium bromide proliferation assay at different time intervals. Similarly, keratinocytes were seeded onto curcumin-containing scaffolds at equal cell numbers and subjected to the MTT assay. The MTT assay was performed in principle, as described above with some adjustments. The cell-seeded scaffolds were rinsed with sterilized PBS to remove nonadherent cells, and 0.5 mL of 0.1 mg/mL MTT in culture medium was added per well. Then, incubation at 37 °C for 4 h was performed until intracellular purple formazan crystals were visible under microscope. MTT was removed, and solubilizing DMSO solution was added for 30 min to 1 h, until cells were lysed, and purple crystals were resolved. Then, the supernatants were transferred to a new plate for the reading of the optical density at the spectrophotometer (PerkinElmer) at 570/630 nm. As positive controls of proliferation of equal numbers of human skin, fibroblasts or keratinocytes were seeded onto plastic and assayed similarly. Moreover, in order to minimize optical density (OD) measurement artifacts deriving from the absorption of the MTT reagents from the porous scaffolds, nonseeded scaffolds were also subjected to the entire procedure, and the corresponding OD was subtracted for the calculation of the OD in question. All cell experiments were performed in triplicate, and the results were expressed as mean ± standard deviation (SD). Unless otherwise stated, one-way ANOVA with post hoc Tukey test was used. A *p*-value < 0.05 was considered statistically significant.

## 3. Results

### 3.1. Characterization of CS Nanoparticles

Since Cur is a hydrophobic and relatively unstable compound, formulating it with nanosized carriers to improve its efficacy for topical treatments is the most common route [5,61]. Cur-loaded CS-NPs were prepared with the ionic gelation method, with a CS/TPP ratio of 3/1. The relatively large concentration of TPP was chosen because it is believed to attract CS better and form stronger intramolecular interactions, resulting in the formation of smaller particles [5]. The average size of the prepared CS-NPs was measured with DLS at about 160 nm, and their zeta potential was +38 mV, both with and without the encapsulated Cur, in agreement with previous studies [5,6,7,46,62]. Size is a parameter that affects both the release profile and the biological performance of nanoparticles. More specifically, particles <200 nm are believed to control the drug release rates and infiltrate cells more efficiently, while zeta potential >+30 mV provides good stability and ability to adhere to negatively charged biological membranes [46,63]. The prepared CS-NPs-Cur possess those favorable properties and are therefore expected to be able to penetrate the top layers of the skin through the appendageal route, as demonstrated by Abdel-Hafez et al. [6]. The NP yield was ~50%, with an entrapment efficiency of 34% and a loading capacity of 3% (Table 1). The small loading capacity could be attributed to the hydrophobic nature of Cur that limits its interactions with the hydrophilic CS and to the large size of the Cur molecule, as it contains two aromatic ring systems containing o-methoxy phenolic groups, connected by a 7 carbon linker. Maghsoudi et al. reported Cur EE values in CS-NPs from 0.4% up to 12% [24], while LC in the study of Md. A. Khan et al. was 5.6% [64].

**Figure 1:**
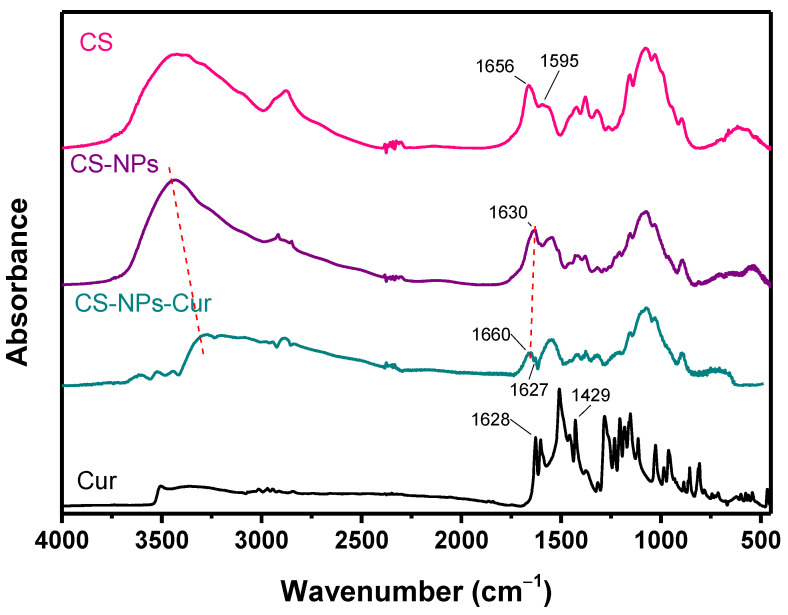
FTIR spectra of CS, CS-NPs, CS-Cur-NPs, and Cur.

**Figure 2:**
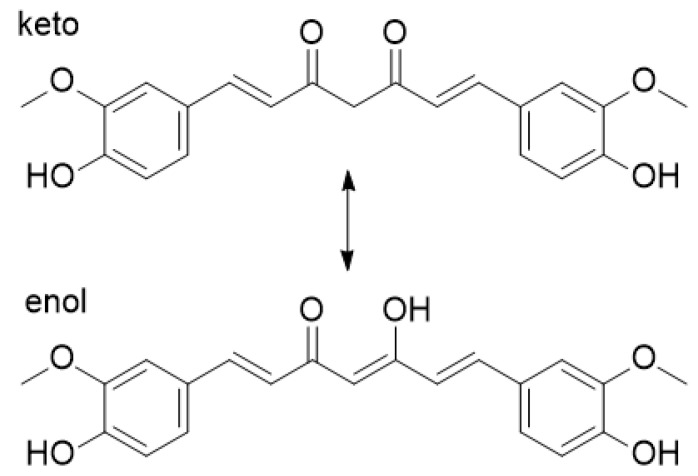
The keto–enol tautomerism of curcumin.

To study the interactions between CS and Cur, FTIR spectra were recorded (Figure 1). Cur exhibits keto–enol tautomerism (Figure 2), with the enol form being the dominant of the two forms [65]. Cur presents characteristic FTIR bands at 3500–3700 cm^−1^ that correspond to hydroxyl stretching vibrations, at 2943 cm^−1^ because of the methylene group stretching vibrations, and at 1628, 1509, and 1602 cm^−1^ from stretching vibrations of C=C and C=O bonds of the aromatic groups. The peak at 1429 cm^−1^ results from the enolic C-OH, and its disappearance in the NPs suggests the presence of electrostatic interactions with the -NH^3+^ groups of CS and/or hydrogen bonding [66]. The peak at 1627 cm^−1^ of the C_ring_-C=C stretch vibration confirms the presence of Cur in the CS-Cur-NPs. The broad adsorption band of the -OH and -NH_2_ groups of CS at 3300–3500 cm^−1^ shifts towards smaller wavenumbers, and its intensity is reduced in the CS-Cur-NPs, while the peak at 1630 cm^−1^ (C=O stretching of amide I) to 1660 cm^−1^ indicates that extensive interactions like hydrogen bonds occurred between the reactive groups of CS and Cur [67].

**Figure 3:**
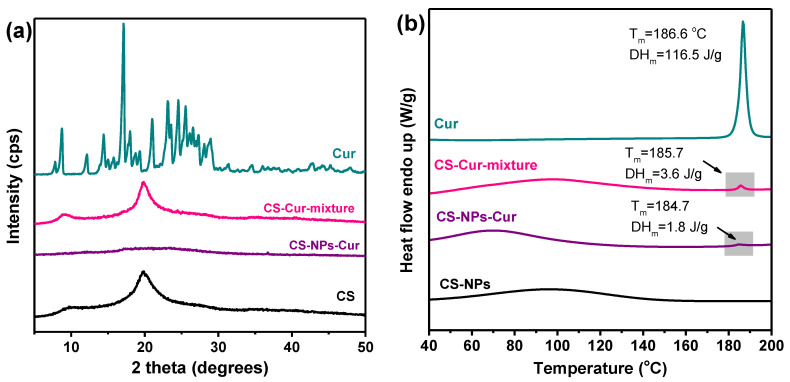
(**a**) XRD diffractograms and (**b**) DSC thermograms of CS, CS-Cur-NPs, and Cur.

DSC thermograms in Figure 3 revealed that only part of the initial Cur amount used showed melting enthalpy, as the heat of fusion of crystalline Cur in the respective DSC thermogram was 1.8 J/g. In contrast, the physical mixture of CS with 3 wt% Cur had heat of fusion of 3.6 J/g. In the XRD diffractograms, no crystalline peaks of Cur appeared either in the CS-NPs-Cur or in the CS-Cur- mixture, likely due to its small amount that is not detectable. The amorphous form of Cur is expected to be more soluble than its crystalline form, which is a highly desired characteristic for hydrophobic active compounds in all drug delivery systems. The presence of interactions between CS and Cur that were concluded by the FTIR spectra might also be the underlying reason for the reduced crystallinity of Cur [5,46,68]. The crystallization of the encapsulated Cur might have also been inhibited by impurities like solvents.

### 3.2. Characterization of the Patches

**Figure 4:**
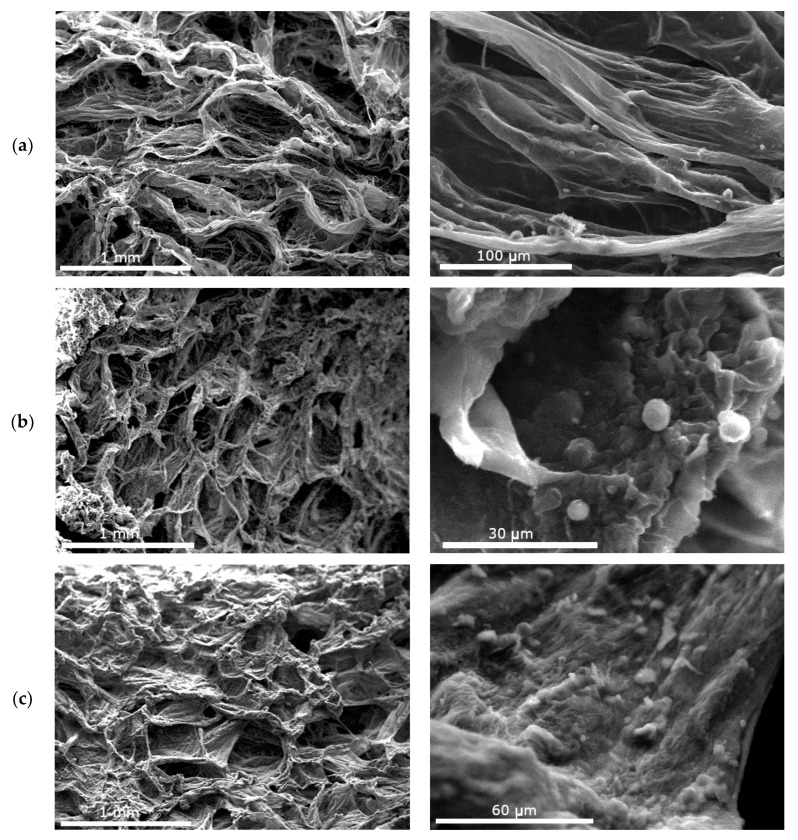
SEM micrographs of the prepared patches in different magnifications: (**a**) Coll-CS Cur, (**b**) Coll-Cross-CS Cur, (**c**) Coll-Cross-Hep-CS Cur.

The morphology of the Coll-based patches was examined with SEM, and the resulting micrographs are presented in Figure 4. The patches have an irregular porous structure that resulted from the sublimation of water during lyophilization, with some visible randomly distributed collagen fibers. The pores are interconnected, a feature that promotes cell proliferation. The pore size range of the Coll-CS Cur patch (Figure 4a) is 150–400 μm. After cross-linking, the Coll-Cross-CS Cur and Coll-Cross-Hep-CS Cur patches show smaller (100–250 μm) more regularly distributed pores, indicative of the more stable structure of the cross-linked patches. All patches possess satisfactory pore size for cell attachment, proliferation, and regeneration of engineered skin tissue [69]. In larger magnifications, spherical particles appear attached on the collagen patches, which could be aggregated CS-NPs.

**Figure 5:**
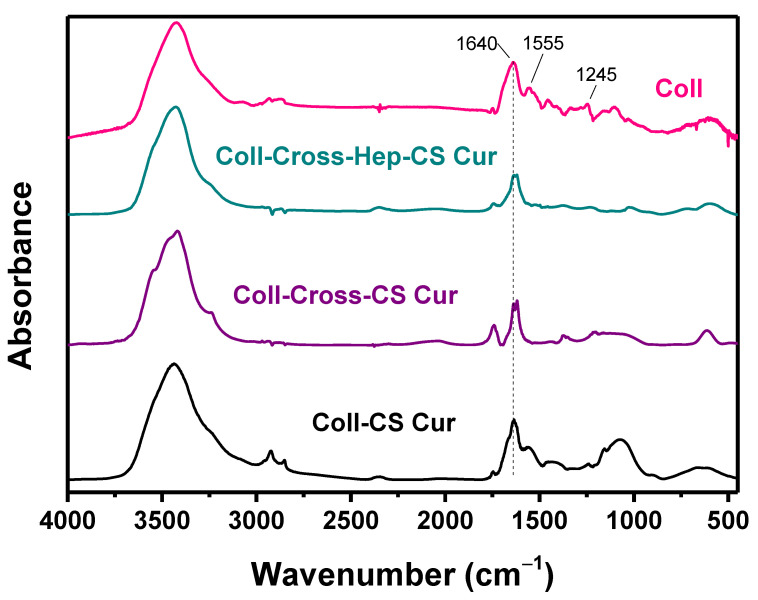
FTIR spectra of collagen patches with CS-NPs.

The FTIR spectra of the Coll patches are presented in Figure 5. In the spectrum of Coll, the main absorption bands that correspond to amide I, amide II, and amide III appear at 1640, 1555, and 1245 cm^−1^, respectively. The broad band at 3000–3700 cm^−1^ corresponds to the amide A N-H stretching vibrations and amide B asymmetrical C-H stretching vibrations.

Cross-linking of proteins with EDC/NHS is a widespread, inexpensive, and nontoxic method that is based on the reaction of reactive carboxyl groups with primary amines. First, EDC forms an active ester intermediate via reaction with the -COOH groups of Coll and/or Hep, which undergoes nucleophilic substitution in the presence of the primary amine groups of Coll and/or CS [70]. Many FTIR bands of CS (Figure 2) appear in similar wavenumbers as Coll, including the O-H stretching vibrations at 3400 cm^−1^, the amide I at 1656 cm^−1^, and the amide II NH_2_ bending vibration at 1595 cm^−1^ [71,72], making it difficult to distinguish these bands between Coll and CS. However, some differences are noticeable between the spectrum of Coll and the spectra of Coll with CS-NPs, mainly in the region 1700–1600 cm^−1^. In the patches with CS-NPs, the amide I band at 1640 cm^−1^ shifts to smaller wavenumbers, suggesting the participation of the C=O group in the formation of hydrogen bonds with the CS-NPs. The band of amide II (1555 cm^−1^) disappears in the cross-linked patches, which is a characteristic of EDC/NHS cross-linked Coll, since the -NH_2_ bonds of both Coll and CS are converted to N-H bonds, confirming the success of the cross-linking reaction [73,74].

**Figure 6:**
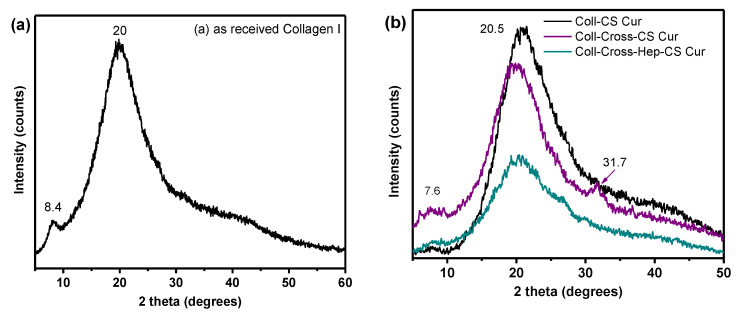
XRD patterns of (**a**) as-received collagen type I and (**b**) collagen patches with CS-Cur NPs.

The structure of Coll in the patches was also examined with XRD, with the resulting diffractograms presented in Figure 6. As-received bovine Coll shows a diffraction pattern typical for native skin Coll [75], with a diffraction peak that indicates the distance between the fibrils at 8.4° and one at 20°, characteristic of the unordered collagen fibrils. The first diffraction peak of Coll in the patches is shifted to 2*θ* = 7.6°, which corresponds to an increase of the distance between the triple helix chains of the fibrils from 1 to 1.17 nm, as calculated by Bragg’s law. The diffraction peak resulting from the triple helical structure of Coll is only visible in the diffractogram of Coll-Cross-CS-Cur at 2*θ* = 31.7° [76]. Overall, the diffractograms suggest that the molecular structure of collagen was maintained in the patches, and no extensive denaturation occurred during their preparation.

**Figure 7:**
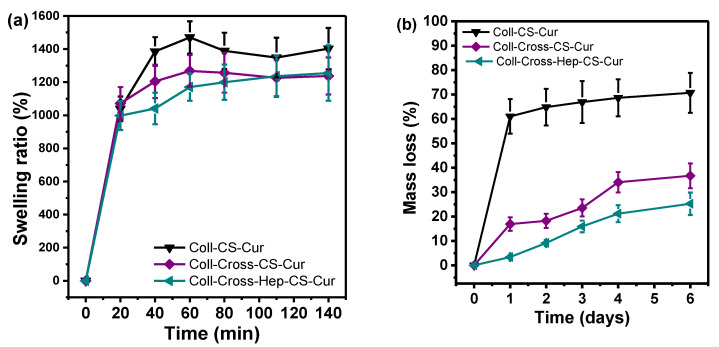
(**a**) Swelling ratio and (**b**) mass loss during the enzymatic hydrolysis of collagen patches with CS-NPs.

The swelling ratios of the patches in SBF are presented in Figure 7a. The capability of a biomaterial to retain water is a crucial parameter, as large swelling ratios relate to increased cell adhesion and proliferation. Especially for psoriatic skin, adequate hydration is essential because the loss of transepidermal water is believed to exacerbate the disease [77,78]. The swelling ratio of the Coll patches in SBF is presented in Figure 7a. Chemical structure, pore size, and shape are factors that affect the ability of a biomaterial to swell. As a very hydrophilic protein macromolecule, Coll can absorb large amounts of water. The swelling ratio of Coll-CS-Cur after ~2 h of soaking is 1400 ± 98%, and is slightly reduced after cross-linking to about 1240% for both cross-linked patches. The reduction is attributed to the smaller pore size and more stable structure of the patches after cross-linking [79,80]. One of the main disadvantages of Coll is that it loses its physical integrity fast because of the large amounts of water it can absorb, and cross-linking makes it more stable and therefore usable as a patch. To further examine the effect of cross-linking on the physical stability of the patches, in vitro enzymatic degradation was studied, and the results are presented in Figure 7b. The enzyme collagenase was used to accelerate biodegradation, and it was expressed as mass loss %. After 6 days of incubation, Coll-CS-Cur lost about 70% of its mass, while after cross-linking, both patches degraded significantly less. More specifically, Coll-Cross-CS-Cur lost 37% and Coll-Cross-Hep-CS-Cur lost 25% of their initial masses. Deceleration of degradation rate is attributed to (i) the masking of collagenase cleavage sites by the cross-links and (ii) reduced swelling ratios of the cross-linked patches [80]. Both swelling ratio and enzymatic degradation tests confirmed the improved hydrolytic stability of the Coll-CS-Cur patches after cross-linking, rendering them suitable for longer-term uses in comparison with neat Coll.

**Figure 8:**
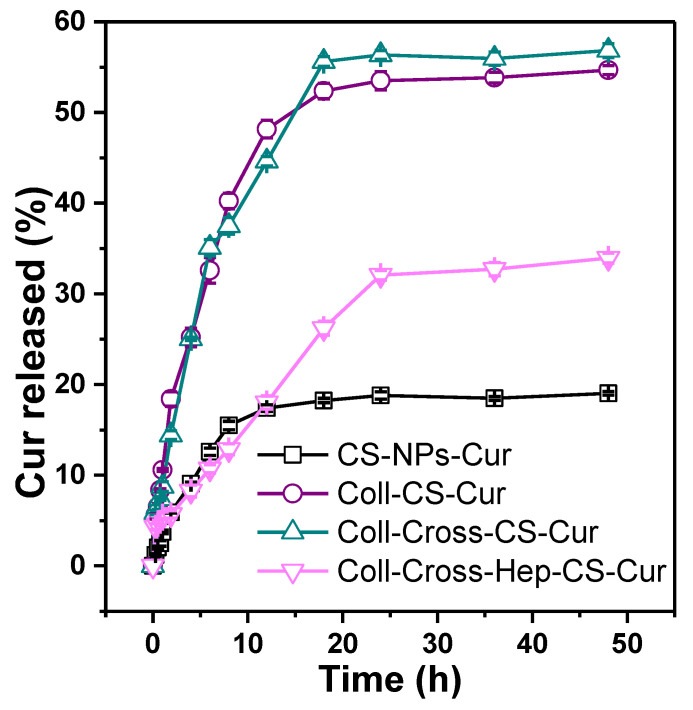
Cumulative Cur release from NPs and collagen patches.

In vitro Cur release was measured in acetate buffer with pH = 5.5 to simulate the pH of the skin. The resulting release profiles are shown in Figure 8. Release profiles depend on several factors, like degradation of the polymers involved, affinity of the drug with the polymers, molecular weight, and physical structure. All samples show a progressive, relatively slow release of Cur that reaches a plateau after 24 h. The release from CS-NPs is fast during the first 10 h, while that fast step lasts for about 20 h for the patches, followed by sustained release, revealing a biphasic process. Burst release is considered an advantage in certain cases, such as wound treatment [81]. The initial burst release is attributed to swelling, porosity, and the presence of free, unbound drug, especially in hydrogel delivery systems [82]. Burst release of Cur encapsulated in gelatin microspheres from collagen–nanocellulose scaffolds was attributed to dissolution of surface Cur on both the scaffold and the microspheres [49,83,84]. The second, slower step could be caused by Cur, which is trapped inside the NPs and has to follow a longer diffusion path [85]. When the CS-NPs were added in the Coll solution during their preparation, the swelling of the NPs might have caused some Cur to be released from the NPs and become dispersed in the Coll hydrogel. This prereleased Cur could migrate during lyophilization and storage via diffusion by convection with the water, resulting in its uneven distribution throughout the patches, with larger amounts of Cur present on their surfaces [81]. About 19% of Cur was released from CS-NPs-Cur. Low Cur % release values from silk fibroin NPs reported by Montalbán et al. were attributed to low drug loading and trapping of Cur inside the NPs rather than on their surface [86]. The low drug loading of the prepared CS-NPs-Cur could be the reason behind the small % release measured. Interestingly, incorporation of CS-NPs-Cur in the Coll patches increased the % release of Cur [87]. This is uncommon because Cur must permeate through both the CS-NPs and the Coll hydrogel, and subsequently, its release would be decelerated. The increase in the release rate of Cur from the patches might be a result of the accumulation of Cur on the surfaces of the Coll patches during lyophilization, the large porosity, and the larger susceptibility of Coll to hydrolysis in comparison with CS-NPs [88]. Such decelerated release of Cur was reported for a synthetic polymer hydrogel containing Cur encapsulated in micelles [89]. When comparing the release from the three patches, Coll-Cross-Hep-CS-Cur releases Cur significantly slower, in agreement with the smaller swelling and degradation rate as well as pore size of the patch, as shown in Figure 7 and Figure 4, respectively.

### 3.3. Cell Studies

**Figure 9:**
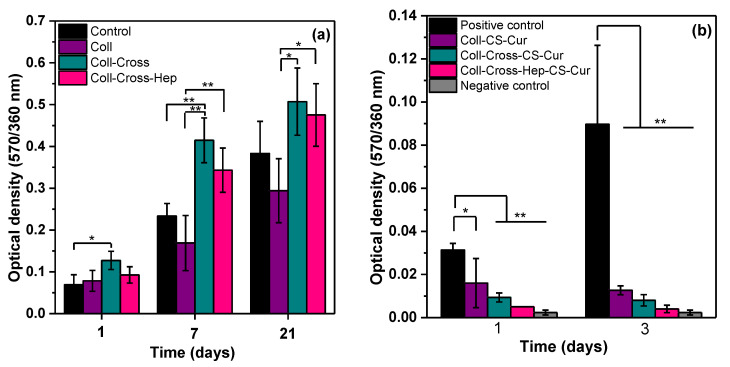
(**a**) MTT assay results on the proliferation of fibroblasts seeded on the Coll patches without Cur. (**b**) MTT assay results on the proliferation of psoriatic keratinocytes seeded on the Coll patches with Cur. Mean ± SD. * *p* < 0.05, ** *p* < 0.01.

The results of the MTT assays on cell proliferation on the patches without and with Cur are presented in Figure 9. All different types of Coll patches supported psoriatic fibroblast growth (Figure 9). Repeated measures analysis revealed a significant time effect between all samples (*p* < 0.01), indicating an increase in cell viability over time. As shown in Figure 9a, cross-linking of collagen I with or without heparin enhances psoriatic fibroblast proliferation rates. This effect is reversed when Cur-NPs are added to the patches. As demonstrated in Figure 9b, when psoriatic keratinocytes come into contact with CS-Cur, their growth is significantly inhibited as compared with the positive control of cells cultured onto plastic, and this effect is independent of each scaffold’s specific structure and/or rate of Cur release. More importantly, inhibition is maintained through time, while viable cells are still detected after 3 days of treatment. The inhibitory effect, in addition to the presence of Cur, could also be a result of the presence on CS, as it was found that some highly deacetylated chitosans (89% deacetylation) can inhibit human keratinocyte mitogenesis [90].

The in vitro cytotoxicity of Cur-CS-alginate NPs towards TNF-α-induced psoriasis-like proliferated HaCaT human keratinocytes was recently reported, and results showed that the NPs significantly decreased the viability of the TNF-α-induced cells and were more effective in reducing their proliferation than free Cur, in agreement with the current study [25].

## 4. Conclusions

In this work, cross-linked and non-cross-linked collagens containing Cur-loaded CS-NPs were fabricated and tested as potential patches for the treatment of psoriatic lesions. The patches possessed an irregular pore size that decreased after cross-linking with EDC/NHS and heparin. Cur was mostly amorphous in the NPs and the patches. Cross-linking slightly reduced the swelling ratio of Coll and reduced its enzymatic degradation rate, thus improving its hydrolytic stability. The in vitro release of Cur occurred in two steps, those of initial burst and sustained release over a period of 2 days. Preliminary cell studies demonstrate that all the curcumin patches have an inhibitory effect on the growth of keratinocytes from patients with psoriasis vulgaris in cell culture in vitro, while a number of viable cells are sustained. Further studies are going to be dedicated to the investigation of the penetration, toxicity, and efficacy of the Cur-loaded patches in three three-dimensional (3D) reconstructed skin models of healthy and psoriatic skin.

## Figures and Tables

**Table 1:** Characteristics of the chitosan nanoparticles (CS-NPs) and Cur-loaded chitosan nanoparticles (CS-Cur-NPs).

Sample	Diameter (nm)	Z-Potential (mV)	Nanoparticle Yield (%)	Encapsulation Efficiency (%)	Loading Capacity (%)
CS-NPs	160 ± 40	38	43.0	-	-
CS-Cur-NPs	164 ± 52	38	46.8	34%	3%
